# Himalayan musk deer (*Moshcus leucogaster*) behavior at latrine sites and their implications in conservation

**DOI:** 10.1002/ece3.8772

**Published:** 2022-04-11

**Authors:** Paras Bikram Singh, Pradip Saud, Zhigang Jiang, Zhixin Zhou, Yiming Hu, Huijian Hu

**Affiliations:** ^1^ Guangdong Key Laboratory of Animal Conservation and Resource Utilization Guangdong Public Laboratory of Wild Animal Conservation and Utilization Institute of Zoology Guangdong Academy of Sciences Guangzhou China; ^2^ Biodiversity Conservation Society Nepal Lalitpur Nepal; ^3^ University of Arkansas Agricultural Experiment Station Arkansas Forest Resources Center University of Arkansas at Monticello Monticello Arkansas USA; ^4^ Institute of Zoology Chinese Academy of Sciences Beijing China; ^5^ University of Chinese Academy of Sciences Beijing China

**Keywords:** behavior, conservation, Himalayan musk deer, Latrine

## Abstract

Elusive species often use latrines which also serves as communication and information hubs. Thus, studying behavior at latrines may provide critical insights into the species’ ecology and behavior. While it is established that musk deer use latrines for defecating, very little is known about the endangered Himalayan musk deer (*Moschus leucogaster*) and their latrines. We examined musk deer behavior from the various video clips lasting 238 min altogether, captured at latrine sites during both breeding and non‐breeding seasons in the Annapurna Conservation Area of Nepal. A total of 428 visits by musk deer and 479 behavioral events by them were captured. We constructed an ethogram to describe musk deer behavior and compared behavior across individuals and sex using parametric and non‐parametric tests. We found that musk deer are crepuscular and nocturnal animals. Both male and female musk deer repeatedly and independently visited shared latrine sites as well as exclusively used latrine sites. The proportion of male musk deer visited latrine sites were significantly higher than that of female musk deer. Hence, male musk deer were found more active (>2 times) than females during both seasons. The most frequently observed behavioral activities at the latrine sites were defecating, sniffing and browsing, followed by scrapping and covering, and ignoring the latrine sites. The defecating and sniffing activity were performed throughout breeding and non‐breeding seasons to establish communication among peers and to claim territory. Based on the behaviors observed at the latrine sites, we can presume that Himalayan musk deer likely use latrines to convey various messages, including personality, maturity, sexual status, and territory marking among conspecifics. These findings of this study can be used for the conservation of musk deer in its natural habitat and replicated in captivity to enhance breeding performance that improves long‐term conservation prospects for this species.

## Graphical abstract

Common behaviors of musk deer, such as repeated defecation, sniffing, scrapping, covering, and ignoring at latrine sites covey various messages including personality, update maturity, and territory marking.

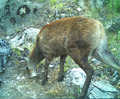

## 1 INTRODUCTION

Musk deer (Moshidae: *Moschus* spp.) are a monogeneric family of small ungulates (7–17 kg) endemic to alpine zones in Asia (Zhou et al., 2004). So far, seven species of musk deer namely *moschiferus*, *chrysogaster*, *leucogaster*, *cupreus*, *fuscus*, *anhuiensis*, and *berezovskii* have been identified (Groves & Grubb, 2011; Singh et al., 2019). All musk deer species are categorized as endangered except *M*. *moschiferus*, the Siberian musk deer, which is classified as vulnerable by International Union for Nature Conservation (IUCN; IUCN, 2012; Nyambayar et al., 2015; Timmins & Duckworth, 2015a,b). The population of this deer is dwindling to the verge of extinction in many regions in its natural range because of poaching for musk pod (Yang et al., 2003). Hence, the conservation status of musk deer is of grave concern.

Musk deer are solitary and territorial, and they exist within a dense forest cover and possess limited vocal repertories (Singh, Saud, et al., 2018). Such characteristics suggest that musk deer rely chiefly on olfaction for communication (Green, 1987). Chemical messages passed among conspecifics (pheromones) and between contraspecifics (allomones) in feces, urine, and in at least three scent glands in males (musk, caudal, and interdigital) are involved in musk deer olfactory communication (Kattel, 1993; Lai & Sheng, 1993).

Musk deer defecate at dedicated locations called latrines. Latrines are often located on or near forest trails, which may facilitate olfactory communication between conspecifics (Attum et al., 2006; Singh, Shrestha, et al., 2018). Several other hypotheses have been proposed to explain latrine‐based communication, including kin recognition (Ramsay & Giller, 1996), transfer of information pertaining to territory or home range boundaries (Boero, 1995), advertisement of reproductive status (Heise & Rozenfeld, 1999), predator–prey recognition (Lewis & Murray, 1993), dispersal (Boero, 1995), and establishing dominance, hierarchies, and social group‐bonds (Gosling et al., 1996). Accumulation of fecal pellets at latrines over time provides evidence of musk deer occupancy of an area (Shrestha & Moe, 2015).

**FIGURE 1:**
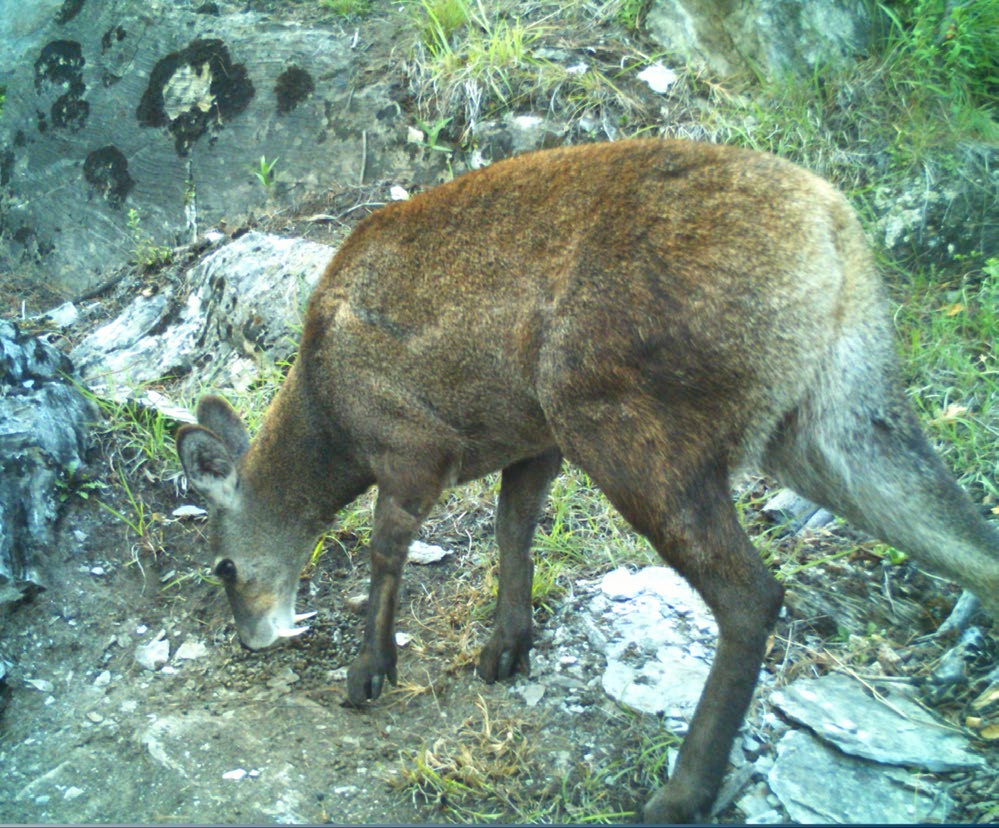
Himalayan musk deer (*Moschus leucogaster*) at latrine site

Many mammalian species possess limited means of acoustic or visual communication, presumably due to their behavior (solitary and nocturnal), habitat (dense vegetative cover), and feeding habits (concentrate feeders). Latrine use has been observed in several species of ungulates, including Oribi (*Ourebia ourebi*; Brashares & Arcese, 1999), Bushbuck (*Tragelaphus scriptus*; Wronski et al., 2006), Dik‐dik (*Madogua kirki*; Hendrichs, 1975), Klipspringer (*Oreotragus oreotragus*; Dunbar & Dunbar, 1974); and in two species of rhinoceroses: the black rhinoceros (*Diceros bicornis*; Linklater et al., 2013) and greater one‐horned Rhino (*Rhinoceros unicornis*; Laurie, 1982). Knowledge of animal behavior can potentially inform us with tools which can be used in wildlife conservation, such as captive breeding, reintroduction, translocation, conservation genetics, reduction of human‐wildlife conflict, and population monitoring (Campbell‐Palmer & Rosell, 2011; Sutherland, 1998). For example, by following reproductive behavioral management strategies in a captive breeding program in China, the giant panda (*Ailuropoda melanoleuca*) population abundance increased threefold in 7 years (Zhang et al., 2004). As a result, the status of the giant panda in China has been improving, and its global status has been reviewed and delisted from endangered to vulnerable (Swaisgood et al., 2004, 2016). In contrast, lack of knowledge of animal behavior can lead to failure to achieve conservation goals. For example, the reintroduction of Oribi (*Ourebia ourebi*) failed in South Africa due to misunderstanding their social behavior (Grey‐Ross et al., 2009). Likewise, African lion (*Panthera leo*) conservation measures taken in Botswana failed because the homing behavior of female lions was underestimated (Soorae, 2018). The population of Himalayan musk deer (Figure 1) requires immediate attention for their long‐term survival. For the long run, conservation of the deer, conservation practices in its range must be more advanced than the traditionally practiced measures that focus on protection. If the behavior of musk deer based on latrine site could be understood, this can be applied to prevent the deer from extinction by managing their habitat and curbing poaching activity.

Little knowledge exists regarding behaviors exhibited by musk deer at latrines. Green (1987) studied the scent‐marking behavior of Alpine musk deer (*M*. *chrysogaster*) in northern India and proposed that defecation was a scent‐marking behavior. This study, however, was limited to the observation of only two male musk deer. Although behavioral studies of captive musk deer have been conducted in China (Meng et al., 2006, 2011; Meng, Cody, et al., 2012; Meng, Li, et al., 2012), caution should be exercised when extrapolating the results from captive‐animal studies to free‐ranging musk deer populations. Considering the limitation of prior studies such as Green (1987), Meng et al. (2011), Meng, Li, et al. (2012) and Meng, Cody, et al. (2012) this research was executed for the first time by installing automated trail cameras in Nepal's greater Himalaya. The objectives of this study were to: (1) describe latrine‐specific behaviors of female and male free‐ranging Himalayan musk deer (*M*. *leucogaster*) during both non‐breeding and breeding seasons, (2) quantify latrine behaviors, and (3) assess the frequency and duration of latrine visits and how the visits varied by sex and season.

## 2 MATERIALS AND METHODS

### 2.1 Study site

**FIGURE 2:**
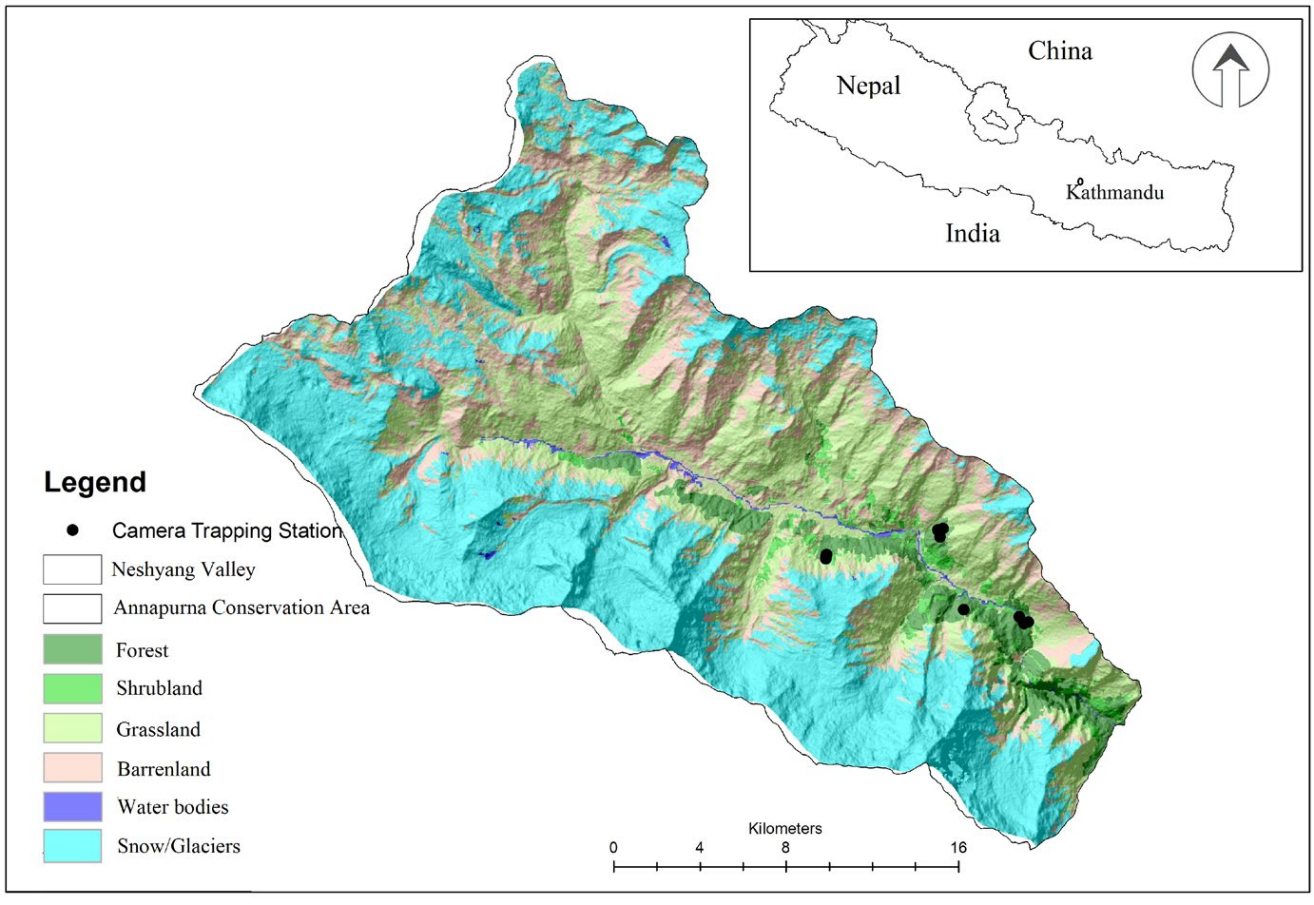
Study area in Annapurna Conservation Area, Nepal

Neshyang valley (690 sq. km) of Manang district which is situated within Annapurna Conservation Area (ACA) was selected as the study site. ACA is the largest protected area in the country which covers an area of 7629 km^2^ extending in five districts of Gandaki Province: Manang, Mustang, Myagdi, Kaski, and Lamjung (DNPWC, 2016; NTNC, 2015). Both Himalayan musk deer (*Moschus leucogaster*) and Kashmir musk deer (*Moschus cupreus*) are found in the temperate and alpine forests of ACA. The distribution of these two species is separated by the Annapurna mountain range. Neshyang Valley, located towards the north of Annapurna mountain range (Figure 2), encompasses an area of 690 km^2^. Four vegetation types, that is, blue pine (*Pinus wallichiana*) forest, Himalayan fir (*Abies spectabilis*) forest, Himalayan birch (*Betula utilis*) forest, and mixed forest are the prime habitat of Himalayan musk deer in Neshyang valley. Mixed forests are comprised of either Himalayan birch and Himalayan fir, or blue pine and Himalayan fir or all three species of trees. A preliminary visit to the forest of Dhikurpokhari (28.597980°N and 84.181286°E), Pisang (28.636689°N and 84.144726°E), Ghyaru (28.637139°N and 84.142526°E), Humde (28.633125°N and 84.091719°E) of Neshyang valley was made to determine whether musk deer latrine sites were present. During our preliminary visits, a total of 15 active latrine sites: three in Humde (HUM), two in Pisang (PIS), seven in Dhikurpokhari (DPK), and three in Ghyaru (GHR) were selected for this study. However, cameras were installed only in 10 active latrine sites (Table 1). Latrine sites which have fresh pellets were considered active latrine sites.

**TABLE 1:** Elevation, slope, aspect, and landcover type of the locations camera traps were placed to record musk deer behaviors at latrine sites in the Neshyang Valley, Annapurna Conservation Area, Nepal, April 2016–January 2017

Camera no.	Elevation (m)	Slope (°)	Aspect (°)	Landcover type
1	3890	28.9	8.9	Dwarf shrubs and sparse herbaceous
2	3627	30.4	26.4	Herbaceous vegetation
3	3948	26.4	324.8	Dwarf shrubs and sparse herbaceous
4	3791	28.0	287.3	Needle‐leaved evergreen forest
5	3978	27.4	305.4	Needle‐leaved evergreen forest
6	3888	27.8	294.6	Needle‐leaved evergreen forest
7	3350	18.1	66.9	Needle‐leaved evergreen forest
8	3145	8.7	135.7	Needle‐leaved evergreen forest
9	3417	15.8	27.7	Needle‐leaved evergreen forest
10	3671	30.1	20.9	Dwarf shrubs and sparse herbaceous

### 2.2 Field work

We selected 10 active sites within habitats of Himalayan musk deer in Neshayang Valley of ACA in Nepal. Camera traps (Cuddeback; Green Bay USA and Bushnell) were installed at each active latrine site (Table 1). Cameras were set on video mode of maximum length to capture video during night and day. Cuddeback can take video of up to 30 s whereas Bushnell can capture video of length 60 s when triggered. Both cameras were set a minimum delay of 10 s. The camera systems were positioned 35 cm above the ground and approximately 2.5 m distance from the latrine sites to capture the entirety of a musk deer and latrine in a single frame. Cameras were set to capture a continuous record of musk deer visits during both day and night (24 h) without any kinds of error throughout the non‐breeding and breeding season. The camera system (pair of cameras in each site) installed during the middle of April 2016 (April 17, 2016) captured the data of animal behavior in non‐breeding season until early August 2016 (August 8, 2016; hereafter, non‐breeding season capture). The same camera systems were operating between the ends of September 2016 (September 19, 2016) and the beginning of January 2017 (January 7, 2017) captured data during the breeding season, October to December (hereafter, breeding season capture). The task of camera installation was completed by April 28, 2016 and September 26, 2016 during non‐breeding and breeding season, respectively. To allow the camera to be fully functional and consistent in the data collection, we considered the video footages of musk deer captured for 90 days (May 1, 2016 to July 29, 2016) in non‐breeding season in our data analysis. Similarly, 90 days between October 1, 2016 and December 29, 2016 were considered in data analysis for the breeding season. Hence, we used a total of 180 days of camera recording in our analysis which excluded time taken for camera installation and uninstallation.

### 2.3 Behavior assessment

We examined all video footages captured in our camera system. Then, the footage of various length (20–120 s) that shows musk deer presence and behavioral activities were clipped. In total, 428 video clips were prepared for data analysis. All detailed information of each video clip, such as date, time, gender, various behavioral activities in time (seconds), visitation length, and the number of visitors was entered in the Microsoft Excel Sheet. Then, an ethogram was used to describe and quantify behaviors. Ethograms which widely used in animal behavior research (Green et al., 2015; White et al., 1998), are catalogs or inventories of various behavior of animals recorded. Each behavior in an ethogram is typically defined to be mutually exclusive and objective to reduce subjectivity. In our case, various behaviors of musk deer recorded and quantified from the videos captured at the latrine sites were used while constructing an ethogram (Table 2). Within each video, ad libitum sampling (Altmann, 1974) was used to record the sex and behavior of each individual musk deer. This sampling method has been used by various researchers, such as White et al. (1998) and Green et al. (2015) to record the behavior of grizzly bear and river otter, respectively. Captured behaviors (i.e., browsing, defecating, ignoring and scrapping, and covering and sniffing) of musk deer were fitted into the ethogram (Table 2).

**TABLE 2:** Ethogram for descriptions of the latrine site behaviors exhibited by free‐ranging Himalayan musk deer (*Moshcus leucogaster*) captured by video in the Neshyang Valley, Annapurna Conservation Area, Nepal, April 2016–January 2017

Behavior	Video	Definition
Browsing	Figure 2, Video 6	Eating or chewing vegetation nearby latrine site
Defecating	Figure 3, Videos 1 and 2	Elimination of fecal matter
Ignoring	Figure 4, Video 4	The musk deer is not responding latrine site. Basically, musk deer ignore latrine site
Scrapping and covering	Figure 5	Use of front legs to move soil, leaves, etc., to cover latrine
Sniffing	Figure 6, Videos 1, 2, and 4	Nose to ground, either while the animal is stationary or walking

### 2.4 Data analysis

Data were analyzed using the R statistical program (R Core Team, 2016). We analyzed the data using parametric approach whenever it meets the assumption, otherwise used non‐parametric, the categorical data analysis approach for the frequency data and contingency table (Agresti & Gottard, 2007). The significance of all statistical tests used in this study was evaluated at α = 0.05 level. Musk deer activity during the time of day was analyzed using the Wilcoxon signed‐rank test since the observed frequency for males and females was paired with the time of the day. To test whether sex and time of the day or sex and season has a relationship with the observed frequency of musk deer by controlling the time of the day, we used the Cochran–Mantel–Haenszel test. The difference in frequency of male and female musk deer visiting each camera system between seasons was analyzed using the Wilcoxon signed‐rank test.

To determine if the observed frequency of males and females was independent of the month and season, we used Chi‐square (χ^2^) analysis for the contingency table. Following the χ^2^ test, we measured the association with Cramer's V statistics to determine if the association was detected between covariate and frequency. The difference in the proportion of latrine site‐visitation of male and female musk deer within the season and month of a season was analyzed using the two‐sample proportion test. Musk deer activities were analyzed using the χ^2^ test and the two‐sample proportion test.

Since the camera systems were deployed in non‐breeding and breeding seasons, it allowed the analyzing of the data within the concept of repeated measurement or pre and post‐treatment frameworks. However, neither the one‐way analysis of variance (ANOVA) model met the normality assumption nor did the non‐parametric approach (Friedman's test) helped to fit the model frequency using the sex and season. Thus, we used paired *t*‐test to determine the mean difference in latrine site‐visitation rates between male and female musk deer considering seasons. Two‐way ANOVA was applied to determine the variation in mean length of the visit by musk deer due to the main and interaction effect of behavioral activity, sex, and season. If the variation in the visit length was significant due to covariates, then the Tukey HSD test was further used to show the mean differences.

## 3 RESULTS

We recorded a total of 428 musk deer visits (Table 3) and a total of 479 behaviors of musk deer (Table 4) between May 1, 2016 and July 29, 2016 (the non‐breeding season) and October 1, 2016 and December 29, 2016 (the breeding season). Approximately 118 min of video during the non‐breeding season and 120 min of video during the breeding season were recorded. In total, cameras were operated for 180 days. Male musk deer visited latrine sites more frequently, for 149 and 114 times respectively during non‐breeding and breeding seasons where female musk deer visited 105 and 60 times respectively during non‐breeding and breeding seasons (Table 3). All latrines sites were visited independently by both males and females.

**TABLE 3:** Frequency of female and male musk deer captured by an individual camera trap during breeding and non‐breeding seasons in the Neshyang Valley, Annapurna Conservation Area, Nepal, April 2016–January 2017

Camera IDs	Non‐breeding season			Breeding season		
	Female	Male	Total	Female	Male	Total
DPK_B	0	4	4	4	19	23
DPK_D	6	12	18	2	12	14
DPK_F	8	41	49	3	42	45
GHR_A	2	0	2	6	3	9
GHR_B	14	31	45	14	9	24
GHR_C	17	3	20	12	1	13
HUM_A	3	46	49	4	15	19
HUM_B	10	0	10	6	4	10
HUM_C	42	7	49	6	0	6
PIS_A	3	5	8	3	9	12
Total	105	149	254	60	114	174,175
Average	10.5	14.9	25.4	6	11.4	17.5
Standard deviation	12.3	17.6	20.2	4.0	12.4	64.7
Coefficient of variation (%)	117.4	118.0	79.7	66.2	108.7	64.7

Note

DPK, Dikhurpokhari; GHR, Ghyaru; HUM, Humnde; PIS, Pisang. Alphabetic letters followed by underscore sign represent the camera ID.

**TABLE 4:** The number of observations (*n*) and percentage (%) of the total female and male musk deer during the non‐breeding and breeding season in the Neshyang Valley, Annapurna Conservation Area, Nepal, April 2016–January 2017

Behavior	Non‐breeding			Breeding		
	Females *n* (%)	Males *n* (%)	Totals *n* (%)	Females *n* (%)	Males *n* (%)	Totals *n* (%)
Browsing	43 (15.1%)	30 (10.6%)	73 (25.7%)	35 (17.9%)	46 (23.6%)	81 (41.5%)
Sniffing	45 (15.8%)	68 (23.9%)	113 (39.8%)	15 (7.7%)	29 (14.9%)	44 (22.6%)
Scrapping and covering	4 (1.4%)	6 (2.1%)	10 (3.5%)	2 (1.0%)	7 (3.6%)	9 (4.6%)
Ignoring	4 (1.4%)	10 (3.5%)	14 (4.9%)	2 (1.0%)	5 (2.6%)	7 (3.6%)
Defecating	24 (8.5%)	50 (17.6%)	74 (26.1%)	8 (4.1%)	46 (23.6%)	54 (27.7%)
Totals	120 (42.3%)	164 (57.7%)	284 (100%)	62 (31.8%)	133 (68.3%)	195 (100%)

Male musk deer average latrine site‐visitation rate of 14.9 ± 17.6 (mean ± standard deviation) and 11.4 ± 12.4, while it was of 10.5 ± 12.3 and 6 ± 4 for female musk deer during non‐breeding and breeding season, respectively (Table 3). Because of the large variability, the paired‐test showed that mean difference in the visitation rate was not significantly different between male and female during non‐breeding (df = 9, *t* = 0.614, *p*‐value = .55) and breeding season as well (df = 9, *t* = 1.173, *p*‐value = .55). Although, the overall musk deer visitation rate was high (25.4 ± 20.2) during the non‐breeding season than of the breeding season (17.5 ± 11.3), the mean latrine site‐visitation rate was not significantly different (df = 9, *t* = 1.35, *p*‐value = .209). However, a large variability (coefficient of variation) around the average visitation rate was observed for male musk deer, female musk deer, and combined musk deer during the non‐breeding season than a breeding season (Table 3). The Friedman rank sum test indicated that the observed frequency of males and females musk deer is not significantly associated with the individual camera system during the non‐breeding (χ^2^ = 0.4, df = 1, *p*‐value = .53) and the breeding season (χ^2^ = 0.01, df = 1, *p*‐value = 1).

### 3.1 Behavior assessment

Browsing, sniffing, scrapping & covering, ignoring, and defecating (see Table 2 for definition) were the behaviors recorded in our cameras system. In total, 479 behavioral events were captured, 284 in non‐breeding season and 195 in breeding season (Table 4). 15.8% of total behavioral events shown by female musk deer were sniffing, and in the case of male musk deer, 23.9% of the total events were sniffing during the non‐breeding season (Table 4). Only 15.1% of behavioral events shown by females were browsing, whereas 10.6% of behavioral events were browsing in the case of male musk deer (Table 4). Out of different behavioral event, 17.6% of behavioral events recorded in camera system was defecation by male which was 8.5% of total event in case of female (Table 4) during non‐breeding season. In contrast, the most common behavior displayed by female musk deer at the latrine sites during the breeding season was browsing (17.9%; Table 4), while both browsing (23.6%) and defecating (23.6%) were equally observed behavioral events in case of male musk deer visiting latrine sites. Sniffing is the second most observed behavior disclosed by females (7.7%) during the breeding season.

Sniffing and defecating by musk deers were observed in a sequence. Most of the time when deer performed sniffing, it was followed by depositing fecal pellets (i.e., defecation) at the latrine site. While approaching the latrine site, musk deer first started sniffing a few feet away from the latrine site and immediately raised their head with alert. Again, the deer sniffed at the latrine site. Musk deer often sniffed once or multiple times (two to three times). After the sniffing event, the deer turn its posterior part on the top of the latrine site. At this position, the deer move its abdomen outside and inside. Then, the deer sat in a semi‐squatting position and defecating by lowering and raising hind part. This process was repeated two to three times (see Videos 1 and 2). Moving abdomen outside and inside might help to squeeze musk at the latrine site. Fecal pellets in latrine sites possess their olfaction to communicate with their peers. Therefore, musk deer were found locating their latrine sites even after the snowfall event. Musk deer covers fresh pellets by leaves or soil or old pellets to preserve odor during the breeding season. Pasting (tail rubbing) behavior (see Video 5) of musk deer was also captured on three occasions. This pasting behavior was not incorporated in the data analysis process, but we have reviewed this behavior based on the videos in the discussion section of this manuscript.

**VIDEO 1:** Sniffing and defecation behavior of female Himalayan musk deer

**VIDEO 2:** Sniffing and defecation behavior of male Himalayan musk deer

**VIDEO 5:** Sniffing and pasting behavior of Himalayan musk deer

## 4 SITE USE

### 4.1 Observation of musk deer at latrine site

**FIGURE 3:**
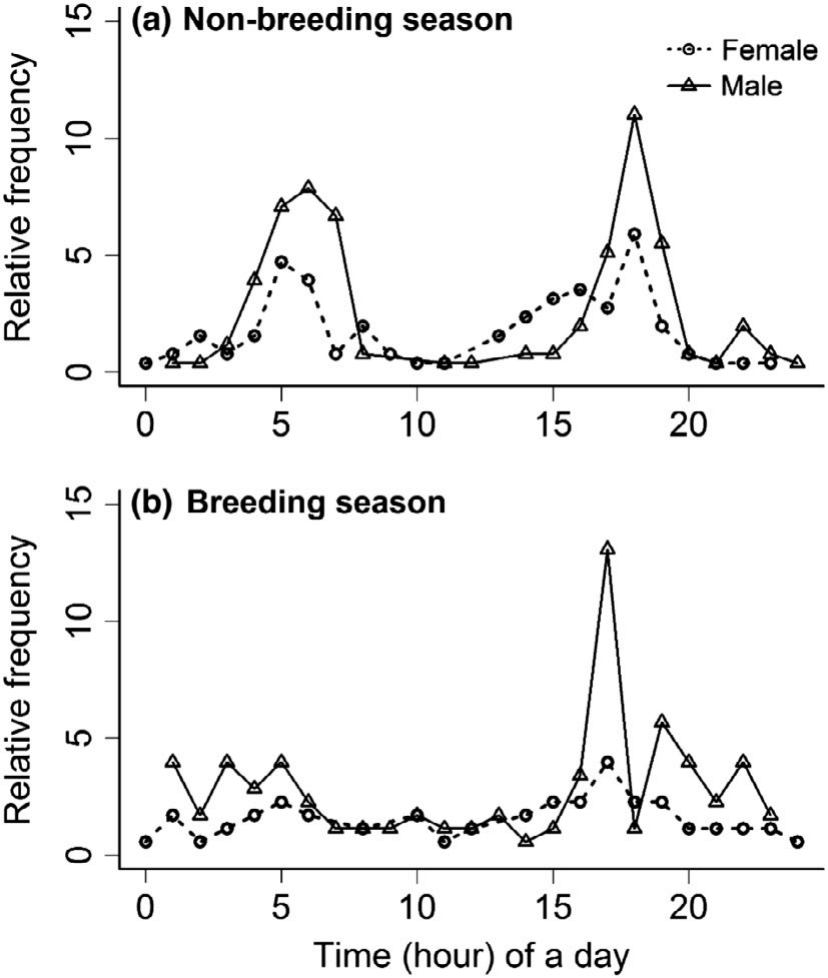
Musk deer captured in camera traps by sex during hours of a day in the breeding and non‐breeding seasons in Manang Nepal, 2016

**FIGURE 4:**
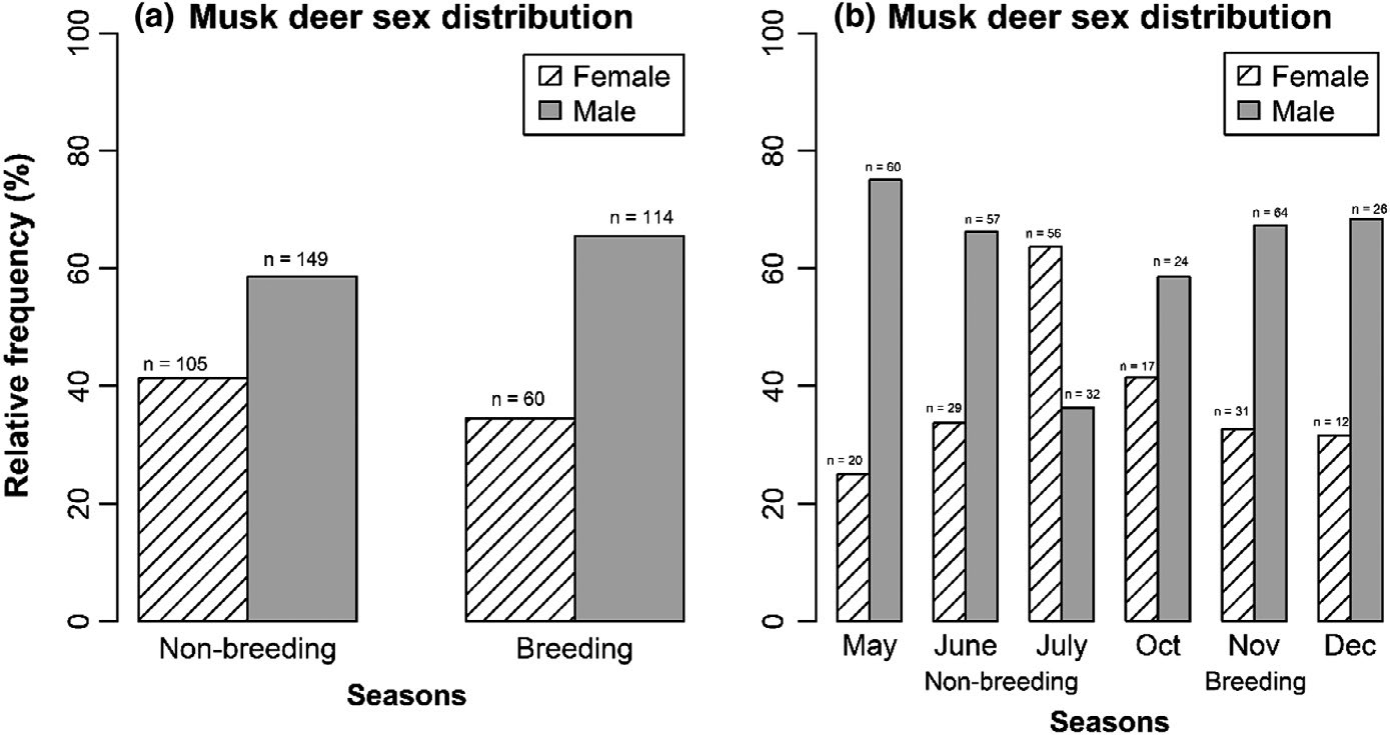
Relative frequencies of male and female musk deer captured by the camera traps during (a) breeding and non‐breeding seasons and (b) during different months in Manang, Nepal, 2016

The relative frequency of male and female musk deer captured at the latrine site during the time of the day for both non‐breeding and breeding season is shown in Figures 3 and 4, and Table 4. The Wilcoxon signed‐rank test result suggests that the captured frequency distributions of males and females were not significantly different (*W* = 225, *p*‐value = .702) during non‐breeding season. However, the captured frequency of males was significantly higher than that of females during the breeding season (*W* = 167, *p*‐value = .046). This result supports that male musk deer are more active than females during the breeding season. The Cochran–Mantel–Haenszel test result showed a significant (*M* ^2^ = 36.56, *p*‐value = .048) relationship between the time of the day and the sex of musk deer when the season was controlled. Thus, it can be inferred that time of the day plays a vital role in both female and male musk deer in their activities. By nature, musk deer are highly vigilant and become active during dawn, dusk, and night. Our results also showed that musk deer (male and female) were found most active during the morning between 3:00 and 8:00 h and during the evening between 15:00 and 20:00 h in both breeding and non‐breeding season (Figure 3a,b).

Male musk deer visited the latrine sites more frequently than female musk deer (Figure 4a). The frequency of visits was independent (χ^2^ = 1.77, df = 1, *p*‐value = .18) of the season. The proportion of male musk deer visited during both non‐breeding season (58.7%) and breeding season (65.5%) was significantly higher (χ^2^ = 14.56, df = 1, *p*‐value < .0001; χ^2^ = 32.29, df = 1, *p*‐value < .0001) than the proportion of female musk deer visited latrine site which were 41.3% and 34.5% in non‐breeding and breeding season respectively. The significantly higher proportion of the male musk deer visiting latrine sites indicates that male musk deer are more frequent visitors than females. Additionally, it suggests that male musk deer are more aggressive than female during the breeding season than females. A plausible reason behind this behavior is that male musk deer have a larger home range and territory than that of females and male may overlap territory of female musk deer. On a monthly basis, male musk deer were found frequently visiting the latrine sites more than female musk deer, except in July (Figure 4b). The relative frequencies of male and female musk deer were dependent on the months during the non‐breeding season (χ^2^ = 28.9, df = 2, *p*‐value < .0001), but it was independent of the months during the breeding season (χ^2^ = 1.17, df = 2, *p*‐value = .56). Overall, the results (Figure 4b) also indicated that male musk deer visited latrine sites more often than female musk deer. Interestingly in October, the early breeding season, the proportion of female musk deer (0.42) visiting latrine sites was not significantly different (χ^2^ = 1.71, df = 1, *p*‐value < .185) than that of male musk deer (0.59).

### 4.2 Various behaviors of musk deer at latrine site

**FIGURE 5:**
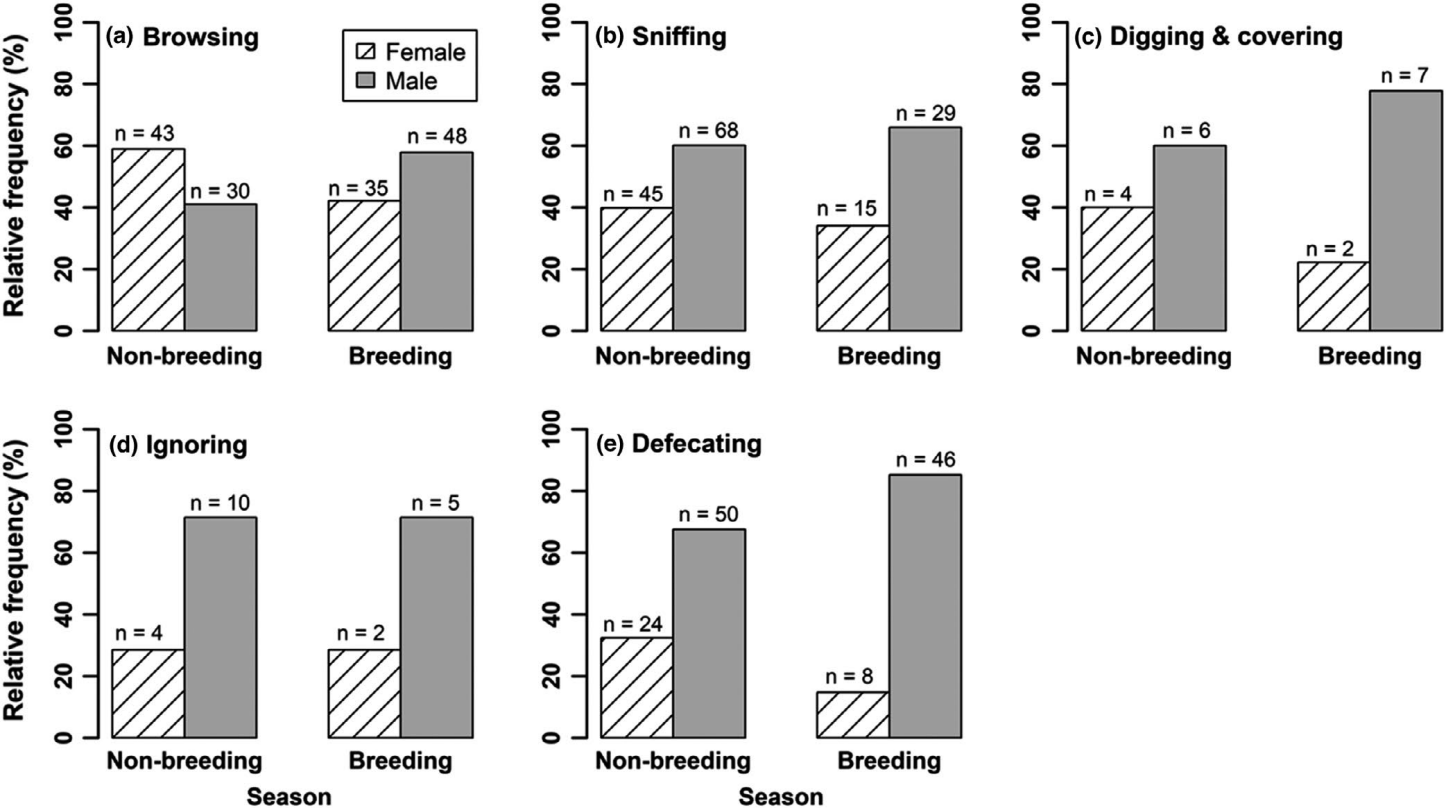
Relative frequencies of different activities of male and female musk deer during breeding and non‐breeding seasons captured by the camera traps in Manang Nepal, 2016

We observed six behavioral activities of musk deer at the latrine sites: defecation, sniffing, scrapping & covering, browsing, ignoring, and pasting (tail rubbing; see Videos 1, 2, 3, 4, 5, and 6). Tail rubbing was recorded close to latrine sites which was noted in only three occasions, so it was excluded from the analysis. The remaining activities were identified as independent events. In both seasons, behaviors such as browsing, defecation, sniffing, scrapping, and covering fresh pellets in the latrine sites were recorded in high numbers for both female and male musk deer (Figure 5). The χ^2^ test of independence indicated that all comparable activities [browsing, sniffing, scrapping & covering fresh pellets, ignoring latrine sites and defecation] of both female and male musk deer were independent (*p*‐value > .05) of the season. From these results, it can be inferred that activities such as defecation, sniffing and scrapping & covering are the activities performed by both males and females at the latrine sites in both breeding and non‐breeding seasons.

**VIDEO 3:** Defending behavior of male Himalayan musk deer in latrine site

**VIDEO 4:** Sniffing and Ignoring latrine site by Himalayan musk deer

**VIDEO 6:** Browsing behavior by Himalayan musk deer

The proportion of males in non‐breeding (0.60) and breeding (0.66) seasons for sniffing activity (see Videos 1 and 2) was significantly higher (χ^2^ = 9.28, df = 1, *p*‐value = .002; χ^2^ = 7.68, df = 1, *p*‐value = .006) than the proportion of females (0.34; 0.43; Figure 5). Both male and female musk deer sniffed at the latrine site to decode various information from the latrine site. Similarly, the proportion of male during the non‐breeding (0.67) or breeding (0.85) seasons for defecating activity were significantly higher (χ^2^ = 16.89, df = 1, *p*‐value < .0001; χ^2^ = 41.79, df = 1, *p*‐value < .0001) than the proportions of female (0.32; 0.15; Figure 5). However, in case of activities such as browsing (see Video 6), scrapping & covering, and ignoring, which was captured in both seasons, the proportion of activities of male was not significantly different from that of female (for all individual activity: χ^2^ < 3.94, df = 1, *p*‐value > .05).

### 4.3 Duration of various activities of musk deer at latrine site

**FIGURE 6:**
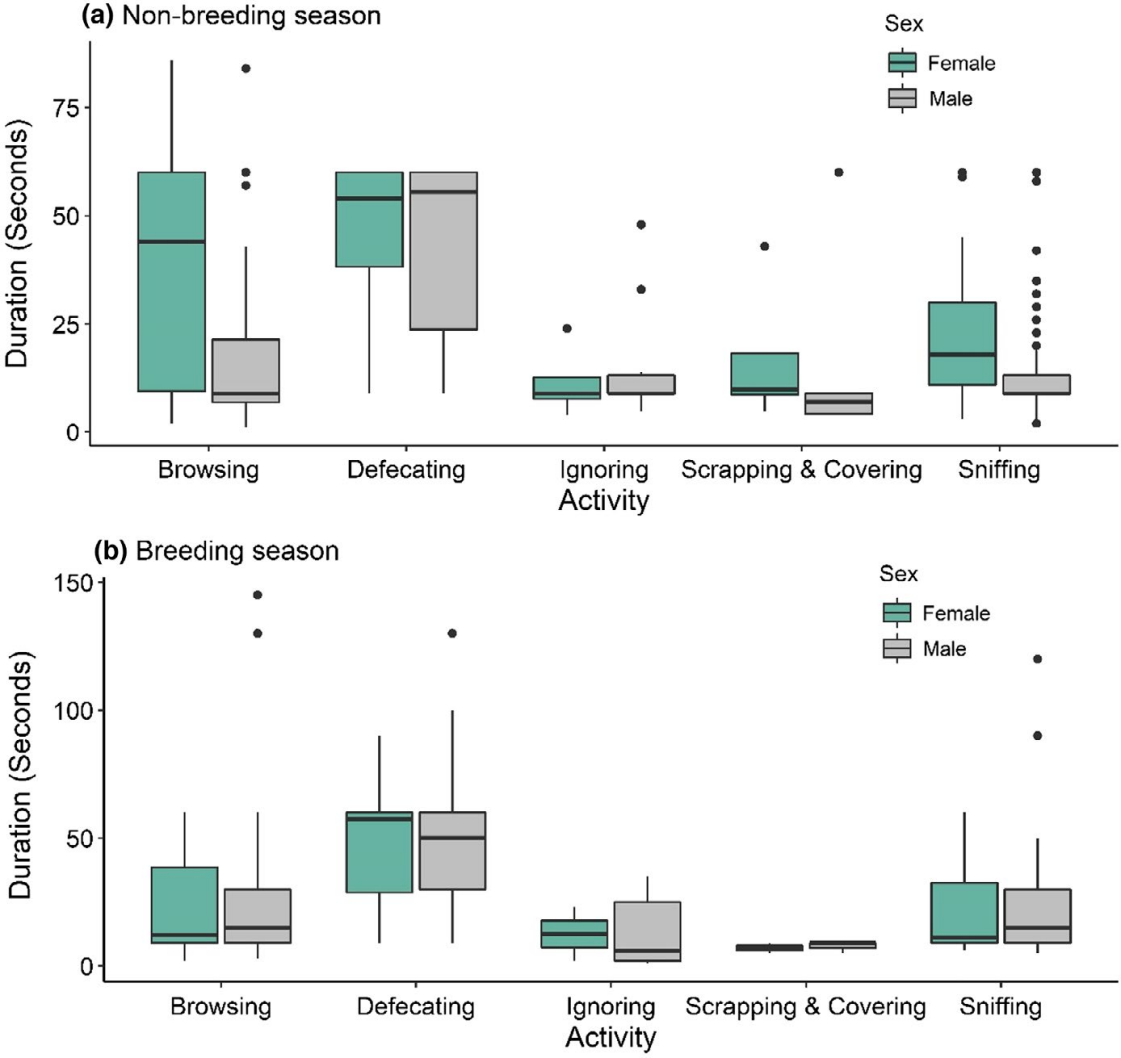
Duration *of various* activities of male and female musk deer captured by the camera traps during (a) breeding season and (b) *non*‐*breeding* season in Manang Nepal, 2016

The distribution of the duration of the visits (in seconds) of musk deer performing different activities at the latrines during the non‐breeding and the breeding seasons is shown in Figure 6. As defecation is the primary purpose of visiting the latrine site, this was observed for a longer durations in both seasons. In contrast, other behavioral activities, such as sniffing, browsing and ignoring were the other behavioral events observed at the latrine site for a shorter durations. During the non‐breeding season, the mean length of visit was the longest for defecating (44.87 ± 19.88 s; mean ± standard deviation), which was followed by browsing (28.9 ± 24.7 s), sniffing (17.2 ± 13.9 s), scrapping & covering (15.9 ± 19.3 s), and ignoring (14.3 ± 12.4 s). During the breeding season, the mean length of visit for defecating was also the longest (51.5 ± 27.9 s) followed by browsing (23.9 ± 25.1 s) and sniffing (23.8 ± 23.7 s), ignoring (13.4 ± 13.9 s), and scrapping & covering (7.7 ± 3.0 s).

The result of ANOVA indicated that the mean length of visits varied among various activities (*F* = 39.63, df = 4, *p*‐value < .0001), sex (*F* = 7.5, df = 1, *p*‐value = .006), and due to the interaction effect of sex and season (*F* = 4.943, df = 2, *p*‐value = .007). Further, the Tukey HSD test suggested that the mean length of visits for defecating was significantly longer than that of scrapping & covering, browsing, ignoring, and sniffing (*p*‐value < .0001). In addition, the mean length of visit for browsing was significantly higher than sniffing (*p*‐value = .022) and scrapping & covering (*p*‐value = .045). Tukey HSD test also suggested that the mean length of the visits of a male during the non‐breeding season was significantly higher than that of a female (*p*‐value < .001), and the mean length of the visit of a male during the breeding season was also higher than that of the non‐breeding season (*p*‐value = .047).

## 5 DISCUSSION

### 5.1 Visit of latrine site

Musk deer visit latrine sites for defecation. However, they make multiple use of it through developing latrine by depositing fresh pellets at some interval of time. Musk deer are solitary. Therefore, male and female musk deer were found visiting the same latrine sites independently and never recorded in pairs. This research also further confirmed that musk deer are both crepuscular and nocturnal animal based on video footage of the deer visiting latrine sites captured in cameras operating throughout 24 h of the day. Observation of musk deer during the daylight (between 8:00 and 15:00 h) is uncommon since the deer rest at their bedding site at this period of time (Singh, Saud, et al., 2018). Either a glimpse of musk deer for a few seconds could be captured, or the sounds of movement through the vegetation could only be heard in the habitat of musk deer. Green (1985) sighted musk deer 74 times in 3 years of their study in Kedarnath Wildlife Sanctuary in India, where the duration of all observations occurred for less than 1 min. Sathyakumar (1994), Vinod and Sathyakumar (1999) sighted musk deer respectively 92 and 65 times during their 3‐year study in India. Singh (2018) encountered the deer seven times during his 2‐year study in Nepal. Even in our video footage, musk deer were found visiting the latrine site cautiously and for a short time with an average duration of 30 s.

We found that single musk deer either male or female solely and repeatedly visited some latrine sites, whereas multiple individuals in different times were recorded at other sites. Such latrine site has been termed as shared latrine sites in this study. Shared latrine sites are visited by both male and female musk deer. However, frequency of visits to such latrine site made by each gender varies with their territoriality. Males visit the latrines predominantly in its locality than females, and vice versa. The movement of both sexes at the shared latrine site helps them to exchange various information. Hence, this research confirms that musk deer visit singled owned and shared latrine site. Green (1987) also suggested similar results to this study, saying that multiple individuals of musk deer may use the same latrine site, that is, shared latrine site where territory overlaps, or single‐owned latrine site.

Overall, male musk deer were seen visiting latrine sites more often (>2 times) than female musk deer. The possible reasons behind male deer movement could be territorial overlap and nature of the male and female deer. Firstly, male musk deer have a larger home range than females, and the territory overlaps with that of females (Kattel, 1993). In the radio collar‐based study, Kattel (1993) found that the home range of the male musk deer (0.22 sq. km) is larger than that of females (0.13 sq. km). Secondly, male musk deer are more territorial than females. And thirdly, male musk deer are more competitive in finding mates and hence, they search a larger radius to find mates. There are some similar findings in other solitary mammals (e.g., Lemur). In the case of Lemur (*Lepilemur leucopus*), the resident males were found visiting latrine sites more often when intruders were moving nearby their territory (Dröscher & Kappeler, 2014).

**FIGURE 7:**
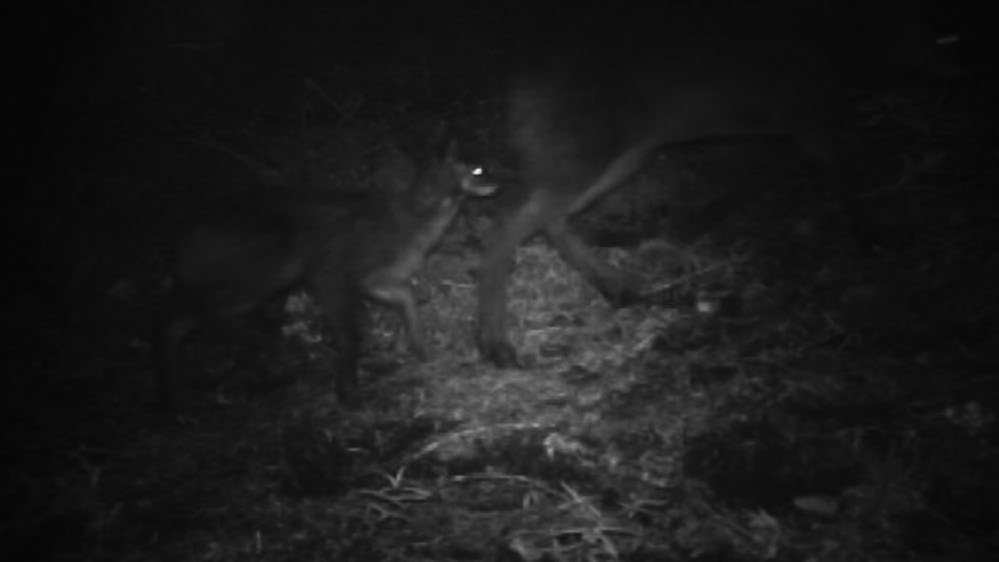
Snapshot of video of female escorting her fawn that was hidden in nearby bushes

In this research, the locations of camera stations were not changed between the two seasons. During the breeding season, it is likely that musk deer might have visited other latrine sites than the latrine sites where trail camera were installed during the breeding season. So, musk deer visits that should be recorded might have not been captured from the same camera locations between breeding and non‐breeding season. Therefore, male and female musk deer were captured less in breeding season than non‐breeding at the latrine site. In July 2016, the visit of female musk deer (Figure 7) was found to be higher than that of the male musk deer. These higher visits were because a female musk deer has hidden newly born fawn (2 months old) in the bushes nearby the camera station. Consequently, that particular female was caught by a camera trap almost every day in July.

### 5.2 Function of latrine site

Defecation is the primary purpose of visiting latrine sites which is highly useful for territorial mammals to communicate with peers, especially in ungulates, primates, and carnivores (Dröscher & Kappeler, 2014). Defecation releases odor (scents) comprising of various chemical signals. The odoriferous substances called pheromones released out of the body of mammals by various means, such as in the form of feces and through caudal glands, and it plays a vital role in communication among conspecifics (Eisenberg & Kleiman, 1972). In the case of species, such as musk deer and bushbuck using waste materials (fecal pellets) for chemical communication is highly economical as they do not require extra energy to release chemical signals, that is, odors in excreta (Wronski et al., 2006). Both male and female musk deer interact while depositing fecal pellets as waste materials at the same latrine site.

**FIGURE 8:**
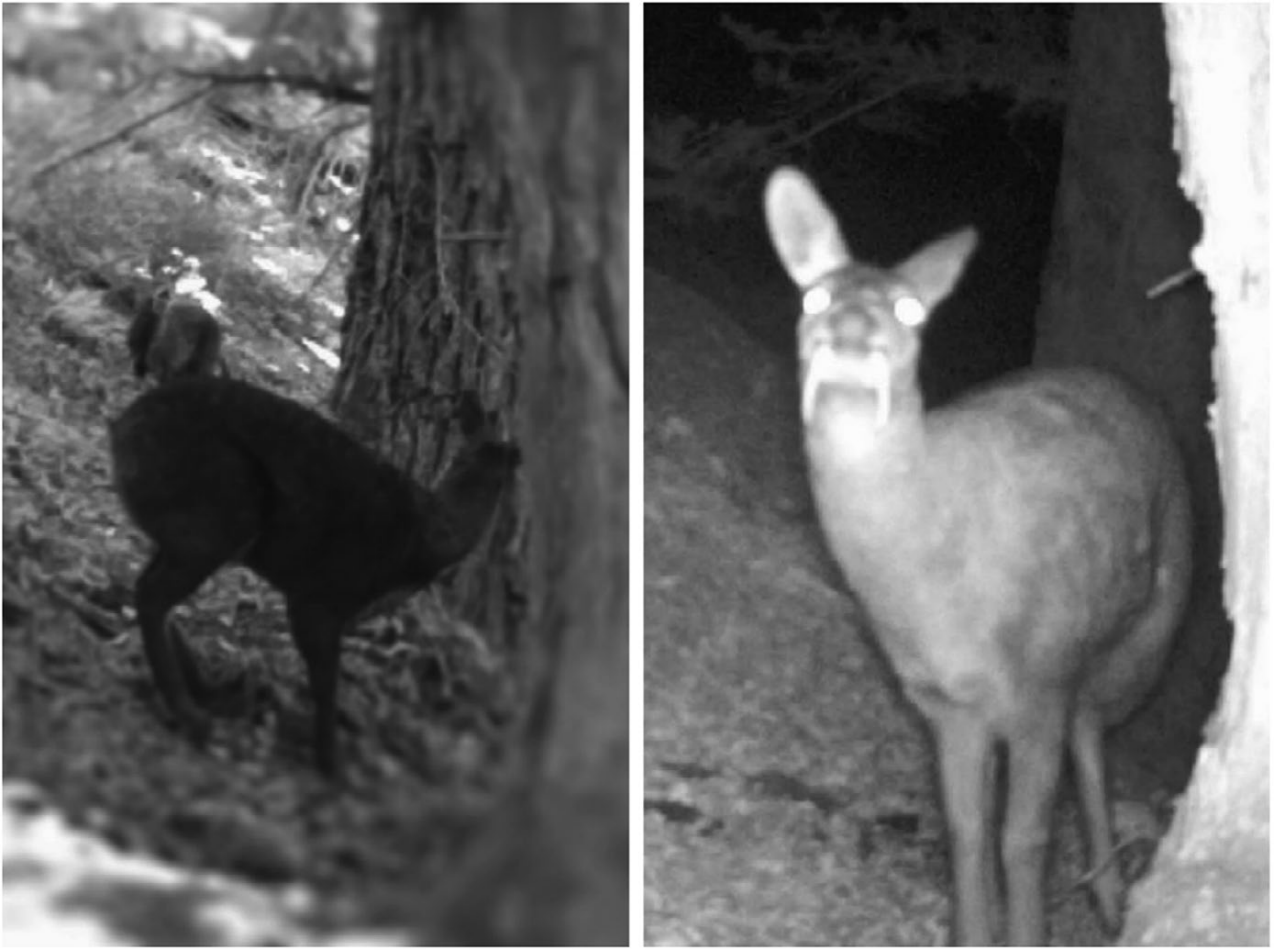
Snapshots of videos of territorial male musk deer showing canines to an intruder

Musk deer latrine sites are highly conspicuous as these sites are established to be detected easily by conspecifics to exchange various information, such as sexual maturity, attracting mate, boldness, and individual identity at the latrine site. The detectability of latrine sites has the advantage of transferring messages contained in the pellets from one individual to another. Therefore, musk deer can be often found defecating at shared latrine sites as well as individually owned latrine sites. Shared latrine sites were found to be used by both males and females (see Table 3) where they may exchange different information such as readiness for mating, warning signs to intruders, and personal status. In the case of using shared latrine sites, each site is dominated by either males or females. Visits of females to the male‐dominated site or vice versa depict that both sexes share various information and update their status. Musk deer also establish single‐owned latrine sites to defend as territory. In other words, territorial musk deer may use single‐owned latrine sites to check whether their territory is being invaded and to alert the intruder to avoid possible confrontation over important resources and mates. These types of strategies are also seen in many other types of ungulates, such as *Gazella arabica* (Wronski et al., 2013), *Oreotragus oreotragus*, Mountain gazelle (*Gazella gazelle*) (Attum et al., 2006), *Ourebia ourebia* (Brashers and Arcese, 1999), and *Tragelaphus scriptus* (Wronski et al., 2006). During this research, on three occasions, male musk deer were found at latrine sites showing canines to the intruder by curling their lips up (Figure 8, Video 3). This behavior is well noticed in musk deer as a territorial behavior (Meng, Cody, et al., 2012; Sathyakumar et al., 2015; Wilson & Russell, 2011).

All other behavioral activities; sniffing, scrapping & covering, and browsing at the latrine site were secondary activities of musk deer. Musk deer sniff at latrines site in every visit. Sniffing activity was found independent of the season, suggesting the activity is performed throughout both breeding and non‐breeding seasons to maintain communication among peers. Even after the snowfall event, musk deer were found sniffing at the latrine site. Locating such sites even after a snowfall showed that musk deer are very precise in finding their latrine site. The possible reason behind locating latrine site precisely by the deer could be due to odors released from the latrine or musk deer could remember location of the latrine site. Green (1987) also found musk deer were sniffing and scrapping at latrine sites covered deeply under the snow. Defecation is one of the means of releasing scents comprising of various chemical signals. Sniffing at the latrine site is a process of receiving such signals. Then, the signals are transmitted to the brain through the vomeronasal organ, which possesses a function of chemical reception. These kinds of functions are well developed in mammals including Artiodactyla (Eisenberg & Kleiman, 1972).

Musk deer were often found ignoring latrine sites (see Figures 5 and 6, Video 4). The possible reasons for ignoring such latrine sites are either (i) the particular deer may be non‐territorial or (ii) the deer was escaping away from other's territory. During our study, we found many times deer were leaving latrine sites after sniffing. In such conditions, deer were seen frightened (see Video 4). The deer visiting their own latrine site were found to be relaxed. Sometimes, musk deer were browsing nearby plants around latrine sites. This study found that sniffing, scrapping & covering, and ignoring were not significantly different behavior between males and females, suggesting both sexes participate in communication through visiting latrine sites. Various activities of musk deer such as developing latrine sites by depositing fecal pellets, visiting both shared and single‐owned latrine sites, accepting and ignoring latrine sites, and defending latrine sites depict multiple function of latrine sites such as claiming territory, defining home range, use of resources and sexual maturity. Visit of latrine sites, sniffing, scrapping, and covering of pellets are used by various mammals for communication which are mostly observed in territorial and solitary mammals (Gosling & Roberts, 2001).

On three occasions, male musk deer were observed rubbing their tail (tail pasting) at vegetation close to the latrine site (see Video 5). Musk deer rubbed underneath of their tail and paste secretion from a caudal gland into the vegetation. In a research conducted in captive breeding center of musk deer, Meng et al. (2011) observed only male deer were found rubbing tail in both seasons whereas females were seen rubbing tail only in the breeding season. Green (1987) also recorded pasting behavior (i.e., tail rubbing) of musk deer in the wild. In another study, Meng et al. (2006) found that tail rubbing only in sexually mature female musk deer. Possibly, rubbing tail by female musk indicates their sexual maturity. In our research, the fewer observations of rubbing tail imply fewer sexually mature female musk deer in the study site during the breeding season.

## 6 IMPLICATION OF LATRINE SITE IN MUSK DEER CONSERVATION

Globally, the population of Himalayan musk deer has been drastically depleted because of poaching to obtain highly valuable musk found only in male musk deer. To comply with their nature (shy, solitary, crepuscular, and nocturnal), musk deer build latrine sites in specific locations to help establish communication with their peers. Though these latrine sites are biologically useful to musk deer, poachers use these sites as a guide to set snares. Snares kill both male and female musk deer indiscriminately, and this method of poaching is causing a dramatic decline in the musk deer population in the wild. To retaliate against poaching, monitoring of the latrine sites can help to trace the movement of musk deer throughout the year. Further, tracing can be helpful to execute an anti‐poaching operation and define suitable habitats of the deer in a particular landscape. Therefore, monitoring latrine sites can serve as the best strategy to curtail poaching, manage habitat, and understand the population status of musk deer.

Currently, most of the viable population of the deer are confined in the protected areas. However, the pressure of poaching within the protected area and habitat degradation due to various anthropogenic pressure cannot be undermined as a potential threat to musk deer. These threats are causing the local extinction of musk deer. In such circumstances, conserving musk deer only in the wild may not guarantee their long‐term survival. Therefore, it would be a good initiative to reproduce a captive population of musk deer while protecting them in the wild. For the success of a captive breeding program, information on animal behavior is the primary requirement. Knowledge of the function and mechanism of the latrine sites used by the musk deer can be helpful to activate mating and maintain captive animal welfare. Notably, understanding the latrine sites, chemical signals, and animal behavior can support and improve the potential conservation disciplines, such as population monitoring, habitat management, anti‐poaching operation, reintroduction, rewilding, conservation farming, and wildlife research.

## 7 CONCLUSION

Musk deer maintain latrine sites by repeatedly defecating in specific locations on the forest trail where odor can be retained for a more extended period. The repeated defecation refresh latrine site with odor. Consequently, odor persists in the latrine site, enhancing the exchange of various messages among conspecifics. We hypothesized that musk deer show various behaviors using latrine sites for different purposes. We used trail cameras for the first time in the higher Himalayas to record the behavior of musk deer at latrine sites. Both male and female musk deer visited latrine sites independently during dawn, dusk, and night confirming these deer as solitary, crepuscular, and nocturnal animals. Interestingly, male and female musk deer shared most of the latrine sites; however, fewer latrine sites were claimed only by individual musk deer, either male or female.

Musk deer visited latrine sites during dawn, dusk, and night throughout the year during breeding and non‐breeding seasons. Frequency and proportion of visit of male musk deer to the latrine sites were higher than that of female musk deer during both seasons. Hence, male musk deer were found more active (>2 times) than female musk deer in both seasons. During the latrine site visits, musk deer exhibited different behaviors, such as sniffing, defecation, browsing, scrapping & covering, ignoring, pasting (tail rubbing), which were captured by our camera system. These behaviors at the latrine sites confirm that musk deer likely use latrine sites for various purposes, such as to indicate readiness for mating to update status (boldness and personality), to warn intruders, and to defend territory. The interaction of male and female musk deer at latrine sites, ownership over latrine sites, and various behaviors shown at latrine sites strongly support that latrine sites are territorial markers and serve as communication centers with their peers.

## CONFLICT OF INTEREST

None declared.

## AUTHOR CONTRIBUTION

**Paras Bikram Singh:** Conceptualization (equal); Formal analysis (equal); Methodology (equal); Resources (equal); Supervision (equal); Writing – original draft (equal); Writing – review & editing (equal). **Pradip Saud:** Formal analysis (equal). **Zhigang Jiang:** Methodology (equal); Supervision (equal). **Zhixin Zhou:** Funding acquisition (equal). **Yiming Hu:** Data curation (equal); Funding acquisition (equal). **Huijian Hu:** Funding acquisition (equal); Methodology (equal); Supervision (equal); Writing – original draft (equal).

## Data Availability

Because of the endangered species and sensitive data nature that could help to locate latrine sites, the data will be made available upon request for the research purpose only.
